# Gene connectivity, function, and sequence conservation: predictions from modular yeast co-expression networks

**DOI:** 10.1186/1471-2164-7-40

**Published:** 2006-03-03

**Authors:** Marc RJ Carlson, Bin Zhang, Zixing Fang, Paul S Mischel, Steve Horvath, Stanley F Nelson

**Affiliations:** 1 Department of Human Genetics, David Geffen School of Medicine at UCLA, Gonda (Goldschmied) Neuroscience and Genetics Research Center, 695 Charles E. Young Drive South, Los Angeles, CA 90095-7088, USA; 2 Rosetta Inpharmatics LLC, 401 Terry Avenue North, Seattle, WA 98109, USA; 3 Cancer Prevention Institute 4100 South Kettering Blvd., Dayton, OH 45439, USA; 4 Department of Community Health, School of Medicine, Wright State University 136 F.A. White Health Center 3640 Colonel Glenn Highway, Dayton, OH 45435, USA; 5 Department of Pathology and Laboratory Medicine, UCLA, 10833 Le Conte Ave. Los Angeles, CA 90095, USA; 6 Department of Biostatistics, UCLA, CHS Suite 51-236 650 Charles E. Young Dr. Los Angeles, CA 90095, USA; 7 Department of Psychiatry, David Geffen School of Medicine, UCLA, 760 Westwood Plaza Los Angeles, CA 90095, USA

## Abstract

**Background:**

Genes and proteins are organized into functional modular networks in which the network context of a gene or protein has implications for cellular function. Highly connected hub proteins, largely responsible for maintaining network connectivity, have been found to be much more likely to be essential for yeast survival.

**Results:**

Here we investigate the properties of weighted gene co-expression networks formed from multiple microarray datasets. The constructed networks approximate scale-free topology, but this is not universal across all datasets. We show strong positive correlations between gene connectivity within the whole network and gene essentiality as well as gene sequence conservation. We demonstrate the preservation of a modular structure of the networks formed, and demonstrate that, within some of these modules, it is possible to observe a strong correlation between connectivity and essentiality or between connectivity and conservation within the modules particularly within modules containing larger numbers of essential genes.

**Conclusion:**

Application of these techniques can allow a finer scale prediction of relative gene importance for a particular process within a group of similarly expressed genes.

## Background

Genes and their protein products carry out cellular processes in the context of functional modules and are related to each other through a complex network of interactions [1]. Understanding an individual gene or protein's network properties within such networks may prove to be as important as understanding its function in isolation [2]. Because of this, numerous studies have focused on the large scale modeling of genomic and proteomic data. Utilizing network theory, these studies have yielded insights into biological systems. For example, both protein interaction networks and gene co-expression networks exhibit a strong modularity reflecting functional partitioning. Both of these network types have been frequently observed as having a scale-free topology, with the existence of highly connected hub nodes [3-7].

Scale-free networks are resistant to random perturbations but sensitive to targeted removal of highly connected nodes [8]. Comprehensive efforts to determine the functional consequences of individual gene deletions in yeast provide the opportunity to study the relationship between individual gene network properties and gene deletion lethality [9]. For example, physical interaction studies in yeast have allowed comparison of connectivity to gene essentiality based on gene deletion. [10]. Typical of scale-free networks, there were few highly connected proteins within the network, and the deletion of a protein with a large number of binding partners is more likely to be lethal in yeast. Thus, the relative position of nodes within a protein interaction network is strongly affiliated with distinct biological properties of individual proteins. Similarly, analysis of unweighted gene co-expression networks have revealed a relationship between connectivity and essentiality across all genes [11].

Correlation of gene expression across a wide variety of experimental perturbations has been shown to cluster genes of similar function [12]. Since this guilt-by-association approach may lead to false positive groupings, approaches have been refined by comparing orthologs across divergent species indicating that highly conserved co-expression is a strong predictor that two genes will function in similar pathways [13-15]. This indicates that functionally related genes are under similar expression constraints. The gene co-expression networks that are based on these relationships have been shown in multiple species to be frequently scale-free and exhibit a small world architecture similar to protein interaction networks even though they are generally more strongly connected [3,14]. It is still unclear however, to what degree the network properties of individual genes within a co-expression network can predict relative gene importance for a particular process. To assess this, we have constructed three networks based on a weighted measure of connectivity [16] of correlated gene expression in yeast using three separate microarray data sets. We assessed relationships between essentiality and connectivity of each gene within the whole network. Further, we define 'modules' (groups of highly correlated genes) and determine that in some instances, the relative importance of genes within these modules can be inferred from network connectivity. We demonstrate that genes which have high connectivity (i.e. 'hub' genes) within a weighted co-expression network are significantly more likely to be essential for yeast viability. Furthermore, we demonstrate a relationship between connectivity and a measure of sequence conservation. Finally, we show that certain critical modules are conserved from one network to another, and that in many cases it is possible to extend the relationship between connectivity and essentiality or between connectivity and conservation within given modules. Thus, analysis of gene co-expression networks provides insight into the functional importance of individual genes within modules of co-expressed genes.

## Results

### Network validation and properties

**Figure 1:**
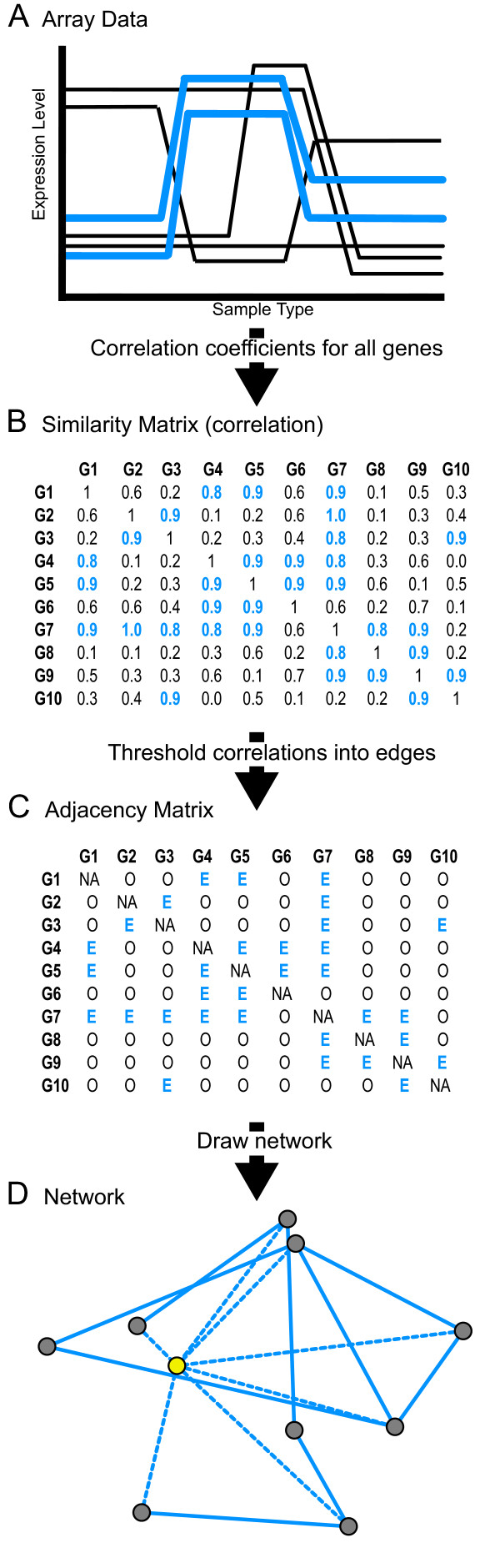
**Generating a gene co-expression network**. (A) Illustration to show how genes that are highly correlated (blue) will look across a dataset. (B-C) Flow chart for defining a gene co-expression network based on a simple Pearson correlation matrix. (D) An example visualization of a network produced by using the Fruchterman Rheingold algorithm on the data in Figure 1B and 1C. The node highlighted in yellow, is an example of the kind of highly connected node that this study shows is more likely to be an essential gene for survival.

In order to test the relationship between gene connectivity within the co-expression network and gene essentiality, we first constructed three networks corresponding to three microarray datasets. The yeast microarray data were derived from experiments designed to analyze DNA Damage (DD) (n = 51), Cell Cycle (CC) (n = 44), and Environmental Response (ER) (n = 151) [17-19]. Each network was constructed as conceptually shown in Figure 1. Nodes were defined as individual genes. The co-expression network is based upon the correlation relationships between individual genes across a dataset (Figure 1A). From the microarray data, the absolute value of the Pearson correlation is the initial measure of gene co-expression similarity (Figure 1B). This co-expression similarity was then transformed into an adjacency matrix (Figure 1C) by raising the similarity to a certain power based on a scale free topology criterion as described previously [16]. The powers selected were 12 (DD), 10 (CC), and 18 (ER). These data can also be visualized as a drawn network (Figure 1D).

Each of the datasets resulted in the construction of distinct networks but with global similarities. The differences in the modularity of the three networks is demonstrated by comparing the heat maps of the topological overlap matrices (Figures 3A, 4A, and 5A). A topological overlap matrix is an indicator of the similarity of all the nodes in question and is defined in detail in the methods section of this manuscript. Approximations of all three of the networks are also drawn in Figures 3B, 4B and 5B using the Fruchterman-Reingold algorithm as implemented in Pajek [20,21] and demonstrate obvious modularity with aggregation of genes into strongly co-expressed groups.

**Figure 2:**
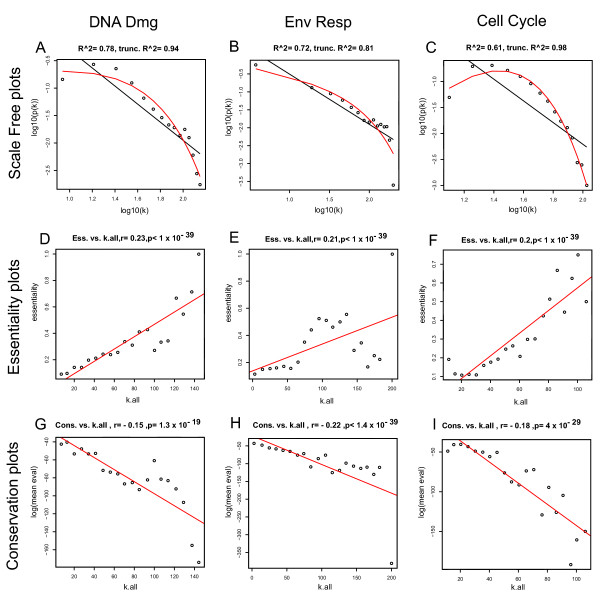
**Global gene co-expression networks**. (A-C) Log-log plots of connectivity distributions in each of the three networks drawn from DNA Damage, Environmental Response, and Cell Cycle, respectively. The linear relationship indicates a scale-free structure. (D-F) Correlation plots showing the relationship between gene group connectivity and essentiality in the same order as A-C. On the y axis of these plots is the percentage of genes determined to be essential in yeast. (G-I) Relationship between the average blastp score of a node and k for all datasets in the same order as A-C. On the y axis of these plots is the average log(e score) of genes within that bin. For plots D-I, the connectivity within each network for each gene was determined and each gene rank ordered by connectivity. 20 equal sized bins for each of the expression datasets were formed and the average connectivity of the genes in each bin plotted on the X axis

Individual gene connections can be difficult to visualize in such complex graphs as presented. Thus, in order to probe the structure of the network, we first sought to determine characteristics of the general network topology. We considered two models. To quantify the fit to the scale free topology model or to the truncated exponential model, we used the model fitting index R-squared of the corresponding linear model involving the log transformed variables [16]. The pattern of network connectivity in these datasets approximated a scale-free topology (Figures 2A, 2B, and 2C). These results are similar to the general trend with genetic perturbation networks and other gene co-expression networks [3,22]. However, gene co-expression networks may better fit an exponential power law [23]. For two of the three constructed yeast networks (DD and ER), the distribution of connectivity (*k*) of genes best fits a power law *p*(*k*) ~ *k*^-*γ *^and the other (CC) best fits an exponentially truncated power law *p*(*k*) ~ *k*^-^γ ^^*e*^-*αk *^[24]. We point out that in general that regardless of the overall structure, the major trend is that the high connectivity genes are few in number, and that most genes have low connectivity (Figure 2A–2C).

### Global network connectivity relationships

Network modeling in biological systems [2,8,11] stresses the relative importance of highly connected nodes. Thus, the small number of high connectivity genes within the gene co-expression networks defined here are expected to be more important. To test the relative importance of each gene across the entire network, we determined the relationship between connectivity and gene essentiality. All the genes for a network were first ranked according to their weighted connectivity and then placed into 20 equal sized bins according to their position in this ranking. The weighted connectivity of a given gene is defined as the sum of its connection strengths with all other genes in the network (see the methods section). The percentage of essential genes in each bin was then plotted against the mean connectivity in each bin (Figure 2D–2F). We also calculated the Spearman correlation coefficient based on the ranked unbinned data and found that there is a relationship between the connectivity and the proportion of essential genes in each of the three datasets (R > 0.2, and p value < 10^-39^) for each network. While the binned plots are well suited for visualizing the results, we calculated the correlation coefficients on the raw, unbinned data to arrive at a valid p-value.

To study whether our conclusions were being unduly influenced by data outliers, we removed genes whose connectivity was more than two standard deviations away from mean connectivity but found no substantial variation in the reported relationships. Thus, there is a clear relationship between connectivity of genes, in these weighted gene co-expression networks, and gene essentiality across the whole network. These results are robust with respect to experimental conditions since the three separate datasets all have the same functional relationship between connectivity and gene essentiality. These results are consistent with a single whole network generated from gene co-expression data and using an unweighted network [11], as well as networks based on protein-protein interactions [10].

Yeast co-expression networks fit an evolutionarily plausible model in which network connection distribution is a natural consequence of gene duplication, mutation and deletions [13]. The emergence of approximate power-law distribution (scale free topology) is intimately linked to the growth of the network in which new nodes are preferentially attached to highly connected already established nodes [2,8]. A direct consequence of this network growth model is that high connectivity 'hub' genes are more likely to be highly conserved across different species. Stated another way, within a gene co-expression network the oldest nodes would be more likely than the newer nodes to be in hub positions. With this in mind, we examined the relationship between connectivity and the relative evolutionary sequence conservation of a node (as evidenced by the average best blastp hit log(e score) against *N. crassa C. elegans, D. melanogaster, H. sapiens, and M. musculus*). High connectivity genes are plotted in Figure 2G–2I relative to the average of the blastp hit in four other eukaryotic genomes. As before, we calculated the Spearman correlation for the ranked, but still unbinned, data and found that there is a relatively weak but highly significant correlation indicating that high connectivity genes are more evolutionarily conserved (R = -0.15, p value = 1.3 × 10^-19^, for the DD network; R = -0.22, p value < 1.4 × 10^-39 ^for ER network; and R = -0.18, p value = 4.0 × 10^-29 ^for the CC network). Our results were robust with respect to the removal of outlier genes (i.e. genes whose connectivity was more than two standard deviations away from mean connectivity). This relationship is similar to that reported for gene perturbation networks [22] and in evolutionarily conserved gene co-expression relationships [14].

### Identification of modules

In order to identify modules, we performed hierarchical clustering on the topological overlap matrix derived from these networks as described in the methods section. In doing so, we identified modules that involve conserved processes for rRNA processing, protein synthesis, and ubiquitination (Table 1) using an EASE analysis [25]. One prediction about network constructions is that certain critically important modules ought to be repeatedly detected in independent datasets. Within the network modules (groups of high connectivity genes within the network) produced by the different datasets for gene ontology enrichment, 3 distinct modules that were common to the 3 different data sets were detected (Table 1 and Figure 6). Comparing the modules that had an enrichment in rRNA processing, protein synthesis and the ubiquitin pathway terms across the DD, CC and ER datasets resulted in a highly significant overlap of 215 genes (hypergeometric distribution p = 3.1e-173). Similarly, the protein synthesis module also had significantly more overlap than expected by chance in which 172 genes were present in all three modules (p = 5.6e-99). The ubiquitin module had the least overlap of common genes (12 genes, p = 3.4e-07) probably because of its smaller size in general.

These three processes are well known to be fundamental processes and have been described before in great detail, and so we decided to investigate the nature of these overlapping regions further. Gene members were selected that were shared within similar functional modules from the three datasets and searched for in the KEGG [26] database. In the ubiquitin pathway, 9 of the overlapping 12 genes were found in KEGG, and all of these were found to be in the same proteasome structure. Since the overlap for this module was unusually small due in part to the very small size of the ER dataset's ubiquitin module, we also looked at the overlap just of the CC and DD ubiquitin modules. Of the 57 genes present in both the CC and DD modules, only 14 were present in the KEGG database. Of those 14 members, all of them were in the same proteasome structure. For the Protein Biosynthesis module, 135 of 172 overlapping members were represented in the KEGG database, and of these, 72 out of a possible 86 were components of the KEGG annotated yeast ribosome. Notably, if the 172 overlapping members were first rank ordered by their average network connectivity, and the bottom quartile removed, then 100% of the remaining members are part of this same yeast ribosome structure, which shows that in this overlap set the high connectivity genes were in fact the members of the yeast ribosome. Finally, the overlap of genes detected in the rRNA modules overlap identified purine and pyrimidine synthesis and RNA polymerase 3 pathways. These pathways dominate this list with 30 out of the 49 identified matches to the KEGG database. Unlike the previous two results however, multiple pathways are found that represent this module. The three network modules discussed all contain large protein complexes. This is in agreement with the recent discovery that there is often a correlation between genes that are expressed together and proteins that bind to each other [27]. However, there is no reason to believe that modules in co-expression networks will always correspond to protein complexes since our module detection method should find any set of genes with high pair-wise correlations. Any experimental perturbation that gives rise to a highly correlated gene set is expected to result in a corresponding network module.

### Intramodular network connectivity relationships

**Figure 3:**
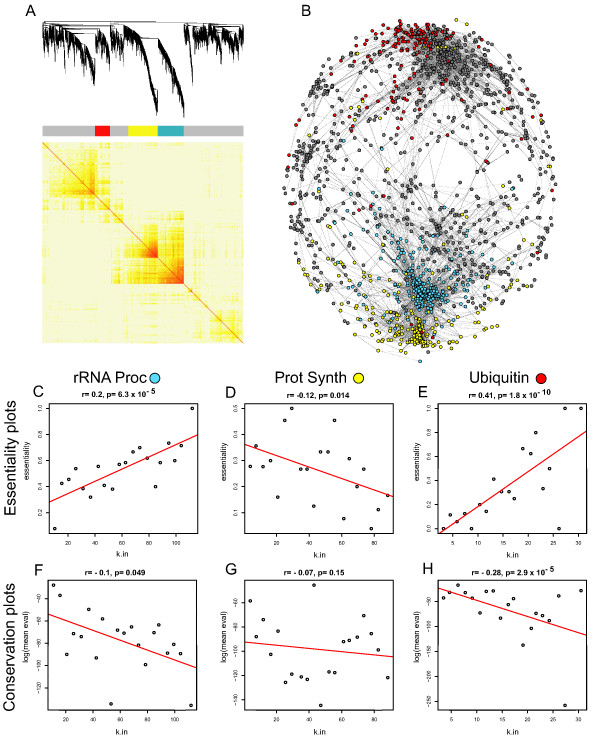
**A co-expression network of the DNA Damage dataset**. For all panels in the figure: blue color represents members of the rRNA processing module, yellow color represents members of the protein synthesis module and red color represents members of the ubiquitin pathway. (A) A hierarchical clustering of the topological overlap matrix for the DD dataset. (B) A drawn network of gene co-expression from the DD dataset. Edges were computed from the Pearson correlation coefficients. Network structure was drawn in Pajek [20]. Each gene is represented as a dot. Edges are drawn as grey lines. Colored dots belong to the module that their color indicates and grey dots indicate all other genes in the network. (C-E) Scatter plots showing the relationship between gene group connectivity and essentiality for rRNA processing, protein synthesis and ubiquitin from the DD network respectively. On the y axis of these plots is the percentage of genes determined to be essential in yeast. The number of essential genes in each module/total number of genes in each module was: 196/390 = 50.3 (C), 122/441 = 27.7 (D), 50/222 = 22.5 (E). (F-H) Relationship between the average blastp score of a node and k for the same module members listed in C-E. On the y axis of these plots is the average log(e score) of genes within that bin. For plots C-H, the connectivity within each module for each gene was determined and each gene rank ordered by connectivity. 20 equal sized bins for each of the expression datasets were formed and the average connectivity of the genes in each bin plotted on the X axis.

**Figure 4:**
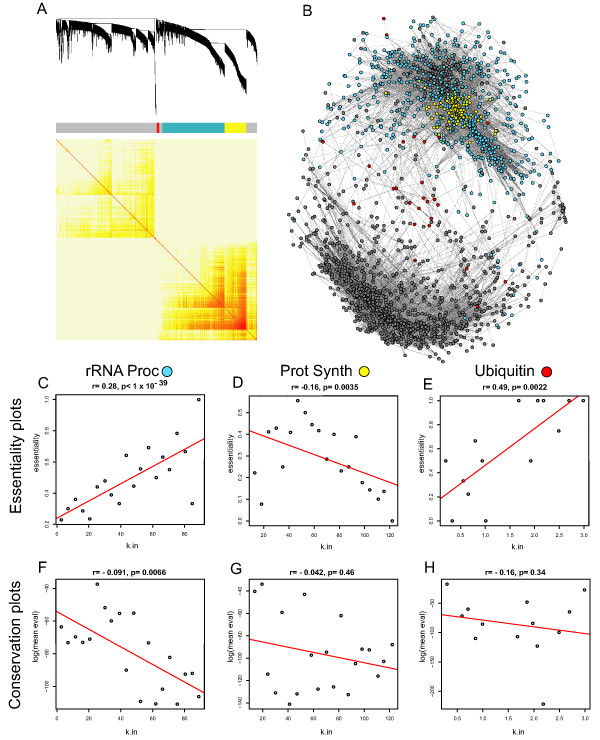
**A co-expression network of the Environmental Response dataset**. For all panels in the figure: blue color represents members of the rRNA processing module, yellow color represents members of the protein synthesis module and red color represents members of the ubiquitin pathway. (A) A hierarchical clustering of the topological overlap matrix for the ER dataset. (B) A drawn network of gene co-expression from the ER dataset. A drawn network of gene co-expression from the DD dataset. Edges were computed from the Pearson correlation coefficients. Network structure was drawn in Pajek [20]. Each gene is represented as a dot. Edges are drawn as grey lines. Colored dots belong to the module that their color indicates and grey dots indicate all other genes in the network. (C-E) Correlation plots showing the relationship between gene group connectivity and essentiality for rRNA processing, protein synthesis and ubiquitin from the ER network respectively. On the y axis of these plots is the percentage of genes determined to be essential in yeast. The number of essential genes in each module/total number of genes in each module was: 317/929 = 34.1 (C), 98/323 = 30.3 (D), 18/37 = 48.6 (E). (F-H) Relationship between the average blastp score of a node and k for the same module members listed in C-E. On the y axis of these plots is the average log(e score) of genes within that bin. For plots C-H, the connectivity within each module for each gene was determined and each gene rank ordered by connectivity.

**Figure 5:**
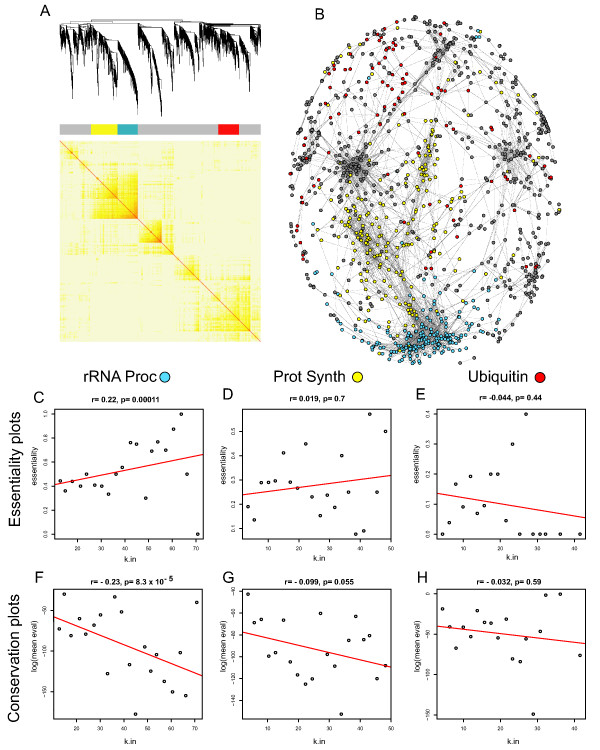
**A co-expression network of the Cell Cycle dataset**. For all panels in the figure: blue color represents members of the rRNA processing module, yellow color represents members of the protein synthesis module and red color represents members of the ubiquitin pathway. (A) A hierarchical clustering of the topological overlap matrix for the CC dataset. (B) A drawn network of gene co-expression from the CC dataset. A drawn network of gene co-expression from the DD dataset. Edges were computed from the Pearson correlation coefficients. Network structure was drawn in Pajek [20]. Each gene is represented as a dot. Edges are drawn as grey lines. Colored dots belong to the module that their color indicates and grey dots indicate all other genes in the network. (C-E) Correlation plots showing the relationship between gene group connectivity and essentiality for rRNA processing, protein synthesis and ubiquitin from the CC network respectively. On the y axis of these plots is the percentage of genes determined to be essential in yeast. The number of essential genes in each module/total number of genes in each module was: 154/300 = 51.3 (C), 105/398 = 26.4 (D), 38/312 = 12.2 (E). (F-H) Relationship between the average blastp score of a node and k for the same module members listed in C-E. On the y axis of these plots is the average log(e score) of genes within that bin. For plots C-H, the connectivity within each module for each gene was determined and each gene rank ordered by connectivity.

**Figure 6:**
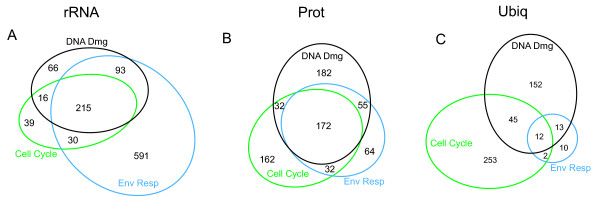
**Module members retrieved from very different datasets are conserved**. For all panels in the figure: black color represents modules retrieved from the DD dataset, blue color represents modules retrieved from the ER dataset, and green color represents modules retrieved from the CC dataset. In each panel A-C a different module is examined for overlap of its members in a Venn diagram. In each diagram the relative overlap of the various modules has been diagramed as different amounts of area with the count represented for each zone by a number that quantifies the precise number of matches.

Having identified 3 functionally similar modules in the three different datasets, we assessed whether these same modules contain the same information as the entire network. Is the connectivity of a gene within the identified modules correlated with the likelihood that a gene was essential by deletion analysis and also whether it was conserved (Figures 3, 4, 5)? To test connectivity correlations within these modules, we define the intramodular connectivity (k.in) as the sum of all connectivity measures only with genes within the same module. Similar to that performed for the total gene connectivity within the whole network, binned values were plotted to observe the possible relationships (Figures 3C–H, 4C–H, and 5C–H). To evaluate significance, Spearman correlation coefficients for the ranked but unbinned data were calculated for relationship of k.in to essentiality and sequence conservation. Interestingly, there was a general trend to similar correlations as that seen within the whole network. The correlation with sequence conservation was generally weak: The ubiquitin module formed from the DD dataset and the rRNA processing modules in the ER and CC were the most correlated with k.in. There was a correlation of k.in and essentiality in all three datasets within the rRNA Processing module (Figures 3C, 4C and 5C), and for two of the datasets (DD and ER but not CC) within the ubiquitin module, and a trend towards a weak anticorrelation within the protein synthesis module (Figures 3D, 4D, and 5D). Thus, some of the relationships between connectivity and essentiality or sequence conservation and not detected when the analysis is restricted within the modules.

### Characterizing modules that contain essential hub genes

The relationship between intramodular connectivity and essentiality is not necessarily universal for any module in any dataset. Whether or not a module contains meaningful information for predicting essentiality depends on the expression data (biological perturbations) that were analyzed. One can view the correlation of k.in and essentiality as a test of the modules importance. The key issue is that in order for a module to demonstrate this relationship, there must be a sufficient number of essential genes within the module. As some modules have substantially fewer essential genes than others, there is little power to detect any relationship with k.in within those modules. We calculated the Spearman correlation between the proportion of essential genes within a module and the correlation of k.in with essentiality and show that there is a strong positive correlation (r = 0.48). Since in actual applications the goal will be to find unknown 'essential' genes, it would be preferable to have another metric of 'essential' gene enrichment. In this regard, it is interesting to note that of the three modules studied in the 3 datasets (9 total), there were 4 that had a correlation of k.in with sequence conservation (p < 0.05). Of these 4 modules, all 4 were shown to have a strong relationship of k.in with essentiality in the same modules. Of the 5 without a correlation (p > 0.05), only one showed a correlation between k.in and essentiality (DD, ubiquitin module). Thus, the detection of a relationship between intramodular connectivity and sequence conservation may predict those modules with a relationship between k.in and essentiality.

## Discussion

Our results extend the inferences made from protein and metabolic networks to the more prolific datasets available from gene expression data. These results are made possible due to broad surveys of expression and the comprehensive characterization of the yeast genome with deletion mutants. In contrast to gene expression data, which are based on the assessment of cells in many different states of perturbation, protein interaction measurements are determined in only a limited set of conditions which by nature only capture a subset of all possible protein-protein interactions. Others [22,28,29] have constructed gene perturbation networks by looking at the expression changes when individual genes are eliminated or in relation to transcription factor expression. Microarray based surveys of gene expression allow detailed surveys of gene expression across a wide variety of experimental perturbations. From these data a strong correlation of gene expression for two genes implies that both genes act within a common functional group and are under similar transcriptional control. Since these networks are not based on direct measurements of protein binding/interaction, the presence of two genes that are highly correlated has a different interpretation than that derived from a protein interaction network. In a co-expression network, two closely tied nodes would be highly correlated but not necessarily direct binding partners. In spite of this key difference, numerous other studies have indicated that genes that are correlated with each other across a modest range of experiments tend to function in similar ways and that this functional categorization is strengthened when gene co-expression exists across large evolutionary distances [12,14].

Here, we use a more general weighted gene co-expression network construction method for network construction that can be applied generically to microarray data using simple correlational analyses of individual genes [16]. Advantages of weighted networks over unweighted networks based on dichotomizing the correlation matrix include a) that the continuous nature of the gene co-expression information is preserved and b) that the results of weighted network analyses are highly robust with respect to the choice of the parameter *β*, [16]. We show that there is a strong correlation with the weighted gene co-expression connectivity measure and gene essentiality and sequence conservation. Our analysis demonstrates that individual conserved modules are identifiable within a network. Moreover, the presence of these modules, which were detected in three independent networks, illustrates that these networks have a structure to them which matches known cellular biology. We have also shown that when sufficient information is available, these correlation modules can provide further information about which of their members are more likely to be important for a given process. The ability to assess whether or not a module is complete enough to be informative using a test like either of the ones employed here will be important for validating the merit of less understood modules in the future.

Our study extends the strong links between the network properties of a gene within co-expression networks and the global importance of each gene to the yeast cell, and may have implications for targeted drug therapy in more complex biological systems like cancer. For instance, one can infer from an analysis of the single gene deletion data that single targets for inhibition can be partially predicted from expression data. However, in biological systems an optimal effect is frequently achieved by inhibiting multiple specific targets. Can we extrapolate network positions and relationships to infer effective combinations of targeted therapeutics? Yeast is an excellent model for establishing the rules for applying network theories to practical predictions. For instance, will double deletions of high connectivity genes within the network which reside in the same module have substantially greater negative consequences to the yeast than double deletions of low connectivity genes? Alternatively, will genes which are connecting distinct modules, and serving as key connections between integrators prove to be better disruption targets? The modeling of such proposed interactions in yeast is attractive because of the relative tractability of yeast studies and the availability of large scale datasets. Technologies like synthetic genetic arrays which automate double deletion screens or chemical genomic profiling provide an expanding capability to survey the effects of multiple target inhibition or deletion [30-32]. However, even with these powerful methods, only a small fraction of the millions of possible combinations will be assayed. For instance in a substantial recent survey of double deletions only 0.02% of all possible double deletions were tested for synthetic lethality [33]. Thus, network based modeling may be a useful adjunct for the design of double knockout experiments or combination inhibition experiments to permit a more efficient search for synthetic lethals. An interplay between predictive models and targeted large scale inhibition studies may be quite useful for surveying pathway analysis in yeast. In the long term, such studies may also be critical in the development of therapies for diseases like cancer.

## Conclusion

In conclusion, our study demonstrates that weighted gene co-expression networks can be used to predict both the likelihood of essentiality and the likelihood of conservation for their gene members within the entire network. Furthermore, we show that these networks can be subdivided into identifiable modules, which themselves can often contain enough individual information to indicate which genes are more likely to be crucial for a particular process. We have indicated a means for testing whether this is true or not. Thus, we have illustrated how this process can be applied to common microarray data for the purpose of predicting both functional assignment as well as the ranking of gene significance in a particular process with which it is found to be associated. We believe that these kinds of predictions will be helpful for identifying gene targets for therapeutics and for directing multi-drug therapies.

## Methods

### Data sources

Yeast microarray data were obtained from the *Saccharomyces *Genome Database in ratio format [34]. In each case, microarray based gene expression analysis was previously reported to have been performed for the analysis of DNA Damage [18], Cell Cycle [17], and Environmental Response [19]. Not all of the data obtained from each datasets listed was used in these experiments. Because missing data is especially confounding for our analysis, arrays where greater than 5% of the gene expression information was missing were removed from our analysis. The exact number of arrays chosen to represent each dataset was: DNA Damage (n = 51), Cell Cycle (n = 44), and Environmental Response (n = 151). Essential genes were determined from the *Saccharomyces *Genome Deletion Project [35]. Genomic data for conducting the blastp searches were obtained from the following locations: *Neurospora *genome data (version 7) was obtained from the Broad Institute [36,37]. *Drosophila *genome data was obtained from the Berkeley Drosophila genome project [38], *C. elegans *genome data was obtained from the wormbase database [39], while both the human and mouse genomes were obtained from the UCSC genome browser [40]. The blastp algorithm was run locally.

### Weighted Network construction

In gene co-expression networks, each gene corresponds to a node. The neighbors of a node *i *are the nodes that are connected to the node *i*. Two genes are connected by an edge with a weight indicating the connection strength. A gene co-expression network can be represented by an adjacency matrix *A *= [*a*_*ij*_], where *a*_*ij *_is the weight of a connection between two nodes *i *and *j*. For all of the networks considered in this paper, the connectivity equals the sum of connection weights being considered.

Our analysis follows a general framework for construction of gene co-expression networks [16]. To transform the co-expression measure (here the absolute value of the Pearson correlation matrix) into measures of pair wise connection strengths, one can make use of an adjacency function. The choice of the adjacency function determines whether the resulting network will be weighted (soft thresholding) or unweighted (hard thresholding). A widely used adjacency function is the signum function which implements 'hard' thresholding involving the threshold parameter tau. Specifically, *a*_*ij *_= *Ind*(|*cor*(*x*_*i*_, *x*_*j*_)| > *τ *where the indicator function takes on the value 1 if the condition is satisfied and 0 otherwise. Hard thresholding using the signum function leads to intuitive network concepts (e.g., the node connectivity equals the number of direct neighbors), but it may lead to a loss of information. For instance, if the threshold has been set to 0.8, there will be no connection between two genes if their correlation equals 0.79. To avoid the disadvantages of hard thresholding, Zhang and Horvath [16] proposed a 'soft' power adjacency function: *a*_*ij *_= |*cor*(*x*_*i*_, *x*_*j*_)|^*β *^with the single parameter beta. The power adjacency function preserves the continuous nature of the gene co-expression information and, leads to results that are highly robust with respect to the choice of beta.

Zhang and Horvath [16] also proposed a criterion for choosing the power beta of the adjacency function which is based on the fact that despite significant variation in their individual constituents and pathways, metabolic networks have been found to display approximate scale free topology. The linear regression model fitting index R^2 can be used to quantify how well a network satisfies a scale-free topology. While the scale free model has only 1 parameter (*γ*), the truncated exponential model allows for 2 parameters *γ *and *α*. Empirically, we find that the two parameters of the truncated exponential model provide too much flexibility in curve fitting so that the R-squared values are always high [16]. Thus, we focus on the scale free topology model fitting index in this application and emphasize the major trend that the high connectivity genes are few in number, and that most genes have low connectivity (Figure 2A–2C). There is a natural trade-off between maximizing scale-free topology model fit (*R^2*) and maintaining a high mean number of connections: parameter values that lead to an *R^2 *value close to 1 may lead to networks with very few connections. These considerations motivated the scale-free topology criterion. To choose the parameters of an adjacency function: Only those parameter values that lead to a network satisfying scale-free topology at least approximately were considered (e.g. signed *R^2 *> 0.80). When considering the signum and power adjacency functions, we find the relationship between *R^2 *and the adjacency function parameter (tau or beta) is characterized by a saturation curve. In our applications, we use the first parameter value where saturation is reached as long as it is above 0.8. An *R^2 *> .80 results in networks that may only approximate the scale free property as is highlighted by the Cell Cycle network in this manuscript.

For each dataset, we first selected the top 4000 most varying transcripts based on the standard deviation. The network adjacency between 2 genes *i *and *j *was defined as a power of the correlation coefficient between the corresponding gene expression profiles *x*_*i *_and *x*_*j*_, i.e. *a*_*ij *_= |*cor*(*x*_*i*_, *x*_*j*_)|^*β *^where the power beta is chosen using the scale free topology criterion. From this function, the 3000 most-connected genes were selected for further detailed network analysis. Using the 4000 most varying transcripts was not very restrictive since the original data are derived from yeast arrays and therefore only contained only about 6000 genes in total. Many of these genes needed to be removed due to high rates (>40%) of missing data for a gene across a data set. By definition, module genes are highly connected with the genes of their module (i.e. module genes tend have relatively high connectivity). Thus, for the purpose of module detection, restricting the analysis to the most connected genes does not lead to major information loss for the key points of this presentation. However, there may be applications where genes with relatively low connectivity are biologically interesting so that gene filtering based on connectivity would lead to information loss. The adjacency matrix was also visualized as a graph and the number of topological properties of this graph examined. Important network derived concepts include: hub (a node with many strong connections to other nodes), topological overlap (the interconnectivity between two nodes), modules (tightly connected subsets of the network), connectivity (the sum of the adjacencies, ie. ki=∑u≠iaiu
 MathType@MTEF@5@5@+=feaafiart1ev1aaatCvAUfKttLearuWrP9MDH5MBPbIqV92AaeXatLxBI9gBaebbnrfifHhDYfgasaacH8akY=wiFfYdH8Gipec8Eeeu0xXdbba9frFj0=OqFfea0dXdd9vqai=hGuQ8kuc9pgc9s8qqaq=dirpe0xb9q8qiLsFr0=vr0=vr0dc8meaabaqaciaacaGaaeqabaqabeGadaaakeaacqWGRbWAdaWgaaWcbaGaemyAaKgabeaakiabg2da9maaqafabaGaemyyae2aaSbaaSqaaiabdMgaPjabdwha1bqabaaabaGaemyDauNaeyiyIKRaemyAaKgabeqdcqGHris5aaaa@3B94@) and intramodular connectivity (the sum of the adjacencies within a module). One potential drawback of soft thresholding is that the network cannot be visualized directly because the number of relationships to display becomes intractable. A soft adjacency matrix specifies pair wise connection strengths and all nodes are connected. To visualize the network with Pajek, one needs to threshold the connection strengths. The resulting dichotomized adjacency matrix can be considered as an unweighted network approximation of the weighted network. Therefore, to make a visual representation, only the strongest correlations (0.9 or greater) were drawn in these renderings. Subsequent to this, networks were then visualized with Pajek [20,41].

### Definition of Topological Overlap and Modularity

Topological overlap of two nodes (genes) reflects their relative interconnectivity. For a network represented by an adjacency matrix *A *= [*a*_*ij*_], *a*_*ij *_∈ [0,1], a well-known formula for defining topological overlap was given by [4], and it can be extended to weighted networks[16]:

ωij=lij+aijmin⁡{ki,kj}+1−aij     (1)
 MathType@MTEF@5@5@+=feaafiart1ev1aaatCvAUfKttLearuWrP9MDH5MBPbIqV92AaeXatLxBI9gBaebbnrfifHhDYfgasaacH8akY=wiFfYdH8Gipec8Eeeu0xXdbba9frFj0=OqFfea0dXdd9vqai=hGuQ8kuc9pgc9s8qqaq=dirpe0xb9q8qiLsFr0=vr0=vr0dc8meaabaqaciaacaGaaeqabaqabeGadaaakeaaiiGacqWFjpWDdaWgaaWcbaGaemyAaKMaemOAaOgabeaakiabg2da9maalaaabaGaemiBaW2aaSbaaSqaaiabdMgaPjabdQgaQbqabaGccqGHRaWkcqWGHbqydaWgaaWcbaGaemyAaKMaemOAaOgabeaaaOqaaiGbc2gaTjabcMgaPjabc6gaUjabcUha7jabdUgaRnaaBaaaleaacqWGPbqAaeqaaOGaeiilaWIaem4AaS2aaSbaaSqaaiabdQgaQbqabaGccqGG9bqFcqGHRaWkcqaIXaqmcqGHsislcqWGHbqydaWgaaWcbaGaemyAaKMaemOAaOgabeaaaaGccaWLjaGaaCzcamaabmaabaGaeGymaedacaGLOaGaayzkaaaaaa@5487@

where, lij=∑u≠i,jaiuauj
 MathType@MTEF@5@5@+=feaafiart1ev1aaatCvAUfKttLearuWrP9MDH5MBPbIqV92AaeXatLxBI9gBaebbnrfifHhDYfgasaacH8akY=wiFfYdH8Gipec8Eeeu0xXdbba9frFj0=OqFfea0dXdd9vqai=hGuQ8kuc9pgc9s8qqaq=dirpe0xb9q8qiLsFr0=vr0=vr0dc8meaabaqaciaacaGaaeqabaqabeGadaaakeaacqWGSbaBdaWgaaWcbaGaemyAaKMaemOAaOgabeaakiabg2da9maaqafabaGaemyyae2aaSbaaSqaaiabdMgaPjabdwha1bqabaGccqWGHbqydaWgaaWcbaGaemyDauNaemOAaOgabeaaaeaacqWG1bqDcqGHGjsUcqWGPbqAcqGGSaalcqWGQbGAaeqaniabggHiLdaaaa@4381@ denotes the number of nodes to which both i and j are connected, and *k *is the number of connections of a node, with ki=∑u≠iaiu
 MathType@MTEF@5@5@+=feaafiart1ev1aaatCvAUfKttLearuWrP9MDH5MBPbIqV92AaeXatLxBI9gBaebbnrfifHhDYfgasaacH8akY=wiFfYdH8Gipec8Eeeu0xXdbba9frFj0=OqFfea0dXdd9vqai=hGuQ8kuc9pgc9s8qqaq=dirpe0xb9q8qiLsFr0=vr0=vr0dc8meaabaqaciaacaGaaeqabaqabeGadaaakeaacqWGRbWAdaWgaaWcbaGaemyAaKgabeaakiabg2da9maaqafabaGaemyyae2aaSbaaSqaaiabdMgaPjabdwha1bqabaaabaGaemyDauNaeyiyIKRaemyAaKgabeqdcqGHris5aaaa@3B94@ and kj=∑u≠jaju
 MathType@MTEF@5@5@+=feaafiart1ev1aaatCvAUfKttLearuWrP9MDH5MBPbIqV92AaeXatLxBI9gBaebbnrfifHhDYfgasaacH8akY=wiFfYdH8Gipec8Eeeu0xXdbba9frFj0=OqFfea0dXdd9vqai=hGuQ8kuc9pgc9s8qqaq=dirpe0xb9q8qiLsFr0=vr0=vr0dc8meaabaqaciaacaGaaeqabaqabeGadaaakeaacqWGRbWAdaWgaaWcbaGaemOAaOgabeaakiabg2da9maaqafabaGaemyyae2aaSbaaSqaaiabdQgaQjabdwha1bqabaaabaGaemyDauNaeyiyIKRaemOAaOgabeqdcqGHris5aaaa@3B9A@. Since *a*_*ij *_∈ [0,1], we find that lij=∑u≠i,jaiuauj≤min⁡(∑u≠i,jaiu,∑u≠i,jauj)=min⁡(∑u≠iaiu,∑u≠jaju)−aij
 MathType@MTEF@5@5@+=feaafiart1ev1aaatCvAUfKttLearuWrP9MDH5MBPbIqV92AaeXatLxBI9gBaebbnrfifHhDYfgasaacH8akY=wiFfYdH8Gipec8Eeeu0xXdbba9frFj0=OqFfea0dXdd9vqai=hGuQ8kuc9pgc9s8qqaq=dirpe0xb9q8qiLsFr0=vr0=vr0dc8meaabaqaciaacaGaaeqabaqabeGadaaakeaacqWGSbaBdaWgaaWcbaGaemyAaKMaemOAaOgabeaakiabg2da9maaqafabaGaemyyae2aaSbaaSqaaiabdMgaPjabdwha1bqabaaabaGaemyDauNaeyiyIKRaemyAaKMaeiilaWIaemOAaOgabeqdcqGHris5aOGaemyyae2aaSbaaSqaaiabdwha1jabdQgaQbqabaGccqGHKjYOcyGGTbqBcqGGPbqAcqGGUbGBcqGGOaakdaaeqbqaaiabdggaHnaaBaaaleaacqWGPbqAcqWG1bqDaeqaaOGaeiilaWcaleaacqWG1bqDcqGHGjsUcqWGPbqAcqGGSaalcqWGQbGAaeqaniabggHiLdGcdaaeqbqaaiabdggaHnaaBaaaleaacqWG1bqDcqWGQbGAaeqaaaqaaiabdwha1jabgcMi5kabdMgaPjabcYcaSiabdQgaQbqab0GaeyyeIuoakiabcMcaPiabg2da9iGbc2gaTjabcMgaPjabc6gaUjabcIcaOmaaqafabaGaemyyae2aaSbaaSqaaiabdMgaPjabdwha1bqabaGccqGGSaalaSqaaiabdwha1jabgcMi5kabdMgaPbqab0GaeyyeIuoakmaaqafabaGaemyyae2aaSbaaSqaaiabdQgaQjabdwha1bqabaaabaGaemyDauNaeyiyIKRaemOAaOgabeqdcqGHris5aOGaeiykaKIaeyOeI0Iaemyyae2aaSbaaSqaaiabdMgaPjabdQgaQbqabaaaaa@8964@. Thus, lij+aij≤min⁡(∑u≠iaiu,∑u≠jaju)
 MathType@MTEF@5@5@+=feaafiart1ev1aaatCvAUfKttLearuWrP9MDH5MBPbIqV92AaeXatLxBI9gBaebbnrfifHhDYfgasaacH8akY=wiFfYdH8Gipec8Eeeu0xXdbba9frFj0=OqFfea0dXdd9vqai=hGuQ8kuc9pgc9s8qqaq=dirpe0xb9q8qiLsFr0=vr0=vr0dc8meaabaqaciaacaGaaeqabaqabeGadaaakeaacqWGSbaBdaWgaaWcbaGaemyAaKMaemOAaOgabeaakiabgUcaRiabdggaHnaaBaaaleaacqWGPbqAcqWGQbGAaeqaaOGaeyizImQagiyBa0MaeiyAaKMaeiOBa4MaeiikaGYaaabuaeaacqWGHbqydaWgaaWcbaGaemyAaKMaemyDauhabeaakiabcYcaSaWcbaGaemyDauNaeyiyIKRaemyAaKgabeqdcqGHris5aOWaaabuaeaacqWGHbqydaWgaaWcbaGaemOAaOMaemyDauhabeaaaeaacqWG1bqDcqGHGjsUcqWGQbGAaeqaniabggHiLdGccqGGPaqkaaa@5490@. Since 1 - *a*_*ij *_≥ 0, we find that *ω*_ij _is a number between 0 and 1. There are 2 reasons for adding 1 - *a*_*ij *_to the denominator in the topological overlap matrix: 1) in this form, the denominator can never be 0 and 2) for an *unweighted *network, one can show that *ω*_*ij *_= 1 only if the node with fewer links satisfies two conditions: (a) all of its neighbors are also neighbors of the other node, i.e. it is connected to all of the neighbors of the other node and (b) it is linked to the other node.

In contrast, *ω*_*ij *_= 0 if i and j are unlinked and the two nodes don't have common neighbors. Further, the topological overlap matrix is symmetric, i.e, *ω*_*ij *_= *ω*_*ji*_. and its diagonal elements are set to 0 (i.e. *ω*_*ii *_= 0). The rationale for considering this similarity measure is that nodes that are part of highly integrated modules are expected to have high topological overlap with their neighbors [4].

It is important to note that the definition of topological overlap matrix (equation 1) was taken from the methods supplement of reference [4] since there is a typo in the primary text. Different definitions of the topological overlap matrix are possible, but we have found empirically that many of these possible generalizations lead to very similar modules [42].

Modules are groups of nodes that have high topological overlap. Module identification is based on the topological overlap matrix Ω = [*ω*_*ij *_defined as (1). To use it in hierarchical clustering, it is turned into a dissimilarity measure by using the standard approach of subtracting it from one (i.e, the topological overlap based dissimilarity measure is defined by dijϖ
 MathType@MTEF@5@5@+=feaafiart1ev1aaatCvAUfKttLearuWrP9MDH5MBPbIqV92AaeXatLxBI9gBaebbnrfifHhDYfgasaacH8akY=wiFfYdH8Gipec8Eeeu0xXdbba9frFj0=OqFfea0dXdd9vqai=hGuQ8kuc9pgc9s8qqaq=dirpe0xb9q8qiLsFr0=vr0=vr0dc8meaabaqaciaacaGaaeqabaqabeGadaaakeaacqWGKbazdaqhaaWcbaGaemyAaKMaemOAaOgabaacciGae8N1dyhaaaaa@32C2@ = 1 - *ϖ*_*ij*_).

In networks involving few nodes, modules can easily be identified by inspecting the network, but for large networks involving hundred of nodes, it is useful to generate a topological overlap matrix (TOM) plot, which we review in the following. A TOM plot provides a "reduced" view of the network that allows one to visualize and identify network modules. The TOM plot is a color-coded depiction of the values of [dijϖ
 MathType@MTEF@5@5@+=feaafiart1ev1aaatCvAUfKttLearuWrP9MDH5MBPbIqV92AaeXatLxBI9gBaebbnrfifHhDYfgasaacH8akY=wiFfYdH8Gipec8Eeeu0xXdbba9frFj0=OqFfea0dXdd9vqai=hGuQ8kuc9pgc9s8qqaq=dirpe0xb9q8qiLsFr0=vr0=vr0dc8meaabaqaciaacaGaaeqabaqabeGadaaakeaacqWGKbazdaqhaaWcbaGaemyAaKMaemOAaOgabaacciGae8N1dyhaaaaa@32C2@], described above.

Based on the TOM based dissimilarity matrix we can use hierarchical clustering to discriminate one module from another. We used a dynamic cut-tree algorithm for automatically and precisely identifying modules in hierarchical clustering dendrogram [43]. The algorithm makes use of the internal structure in a dendrogram and clips branches of the dendrogram. The algorithm is based on an adaptive process of cluster decomposition and combination and the process is iterated until the number of clusters becomes stable. No claim is made that our module construction method is optimal and future research should compare different method for cluster identification. Each module represents a group of genes with similar expression profiles across the samples and the expression profile pattern is distinct from those of the other modules. In each case, the modules were then assessed for enrichment in ontology terms based on Fisher's exact test as described in [25]. These modules and their relative connectivities were then separated out for further analysis.

Based on the network modules, we can define the intramodular connectivity (k.in) for each node i (i.e., the number of neighbors in the same module as node i). The connectivity of node i across the whole network is denoted as k.all.

### Blastp

The translated sequences of the entire yeast genome were each blasted against the entire translated sequences of the Human, Mouse, Fly, *Neurospora *and *C. elegans *genomes. In each case, the best hit was determined using blastp, and the *log*(*e score*) scores were averaged across the 5 different comparisons in a manner similar to what was published by [22]. These average blastp scores were then averaged for each point in (Figure 2H–2K, 3F–3H, 4F–4H, 5F–5H). This method is not necessarily an optimal measure of gene conservation, and it is used here simply as an approximate measure of gene conservation, that is independent of annotation. Future research should study the advantages and disadvantages of using different measures for gene conservation in network analysis.

### Analysis of module overlap

We used a hyper-geometric p-value to evaluate the significance of the overlap of the module genes across the 3 data sets. Suppose N1, N2 N3 out of N genes are in rRNA processing module of the DNA Damage data, the Environmental Response data, and the Cell Cycle data, respectively. We then denote by 'overlap' the number of genes in the rRNA module that overlap in the 3 data sets.

To arrive at an upper limit of the p-value, we first computed pair wise p-values as follows. Denote as *p12 *the p-value that by randomly selecting *N2 *genes from a pool of *N *genes with *N1 *module 1 genes, ≥ overlap selected genes are in the rRNA-processing module in both data sets. This p-value can easily be computed using the hypergeometric distribution (dhyper function in R). *N1 *and *N2 *were then swapped to obtain a second p-value, which we denoted as *p21*. The maximum of those two values can be used to assess the significance of overlap between data sets 1 and 2. Similarly, we computed a p-value of overlap between data sets 1 and 3, and data sets 2 and 3. The minimum of the 3 resulting p-values provided an upper limit for assessing the significance of overlap between all 3 data sets, i.e. the final p-value was computed as p = min(max(*p12*,*p21*), max(*p13*,*p31*), max(*p23*,*p32*))

### Data and software

Prepared Data and R code are available online [44].

## Abbreviations

CC, cell cycle; DD, DNA damage; ER, environmental response

## Authors' contributions

MC and SFN conceived of and developed the project, and wrote the final manuscript. MC analyzed the data, participated in the construction of all the software tools used in this study and drafted the manuscript and figures. SH, BZ authored the majority of the R code needed for developing these weighted networks, developing network measures, and providing crucial statistical guidance for this project. ZF helped with the statistical analysis. PM and SFN both contributed key ideas and suggestions as the project progressed. All authors participated in the development of the ideas presented in this manuscript. All authors read and approved the final manuscript.

## Figures and Tables

**Table 1:** Identifying modules that repeated from one dataset to another

**Ontology term**	**rRNA**			**Prot**			**Ubiq**		
	
	**DNA Dmg**	**Env Stress**	**Cell Cycle**	**DNA Dmg**	**Env Stress**	**Cell Cycle**	**DNA Dmg**	**Env Stress**	**Cell Cycle**
35S primary transcript processing	*1E-17*	*8.8E-16*	*7.5E-16*	0.17	0.51	0.52	0.26	1	0.16
processing of 20S pre-rRNA	*2E-32*	*5E-19*	*3.8E-32*	0.018	0.37	1	0.17	1	0.11
ribosomal large subunit biogenesis	*9.6E-13*	*0.0000071*	*3.5E-15*	0.38	1	0.38	1	1	0.62
rRNA modification	*2.1E-08*	*0.0043*	*0.00014*	0.66	0.019	0.00066	1	1	0.62
rRNA processing	*1.2E-25*	*1.6E-16*	*2.5E-23*	0.48	1	0.62	0.11	1	0.046
protein biosynthesis	0.0001	1.1E-11	0.0023	*5.9E-83*	*5.6E-108*	*1.4E-80*	0.53	0.42	0.00033
translational elongation	0.62	0.14	0.63	*2.1E-08*	*3E-08*	*1.7E-09*	1	1	0.64
translational initiation	0.19	1	0.025	*0.000017*	*1.8E-14*	*3.4E-07*	0.41	1	0.25
ubiquitin-dependent protein catabolism	0.039	0.02	0.071	0.027	0.048	0.018	*2.5E-25*	*7.3E-25*	*6.3E-17*
									
ATP-dependent RNA helicase activity	*7.2E-11*	*9.2E-08*	*4.1E-11*	0.39	0.62	1	1	1	0.62
DNA-directed RNA polymerase activity	*6.1E-13*	*4E-08*	*1.4E-09*	0.77	0.012	0.13	0.42	1	0.4
snoRNA binding	*1.3E-27*	*7.6E-17*	*2.4E-25*	0.042	0.51	0.76	0.42	1	0.16
structural constituent of ribosome	0.00012	2.2E-11	0.000078	*3.1E-92*	*5.3E-117*	*1.8E-95*	0.097	0.41	0.0021
translation initiation factor activity	0.16	0.48	0.14	*4.7E-08*	*5.5E-17*	*2.3E-10*	1	1	0.39
endopeptidase activity	0.25	0.21	0.62	0.16	0.62	0.39	*9.2E-21*	*2.6E-18*	*2.7E-11*
									
nucleolus	*1.6E-48*	*3.4E-29*	*1.3E-45*	0.18	0.65	0.84	0.021	0.48	0.0056
nucleus	*1.2E-18*	*8.3E-13*	*7.8E-10*	0.0053	2.1E-07	0.12	0.0048	1	0.033
small nucleolar ribonucleoprotein complex	*7.9E-20*	*2.8E-09*	*1.3E-14*	0.16	0.67	0.42	0.63	1	0.39
cytosolic large ribosomal subunit	0.03	0.0000016	0.0087	*2.1E-65*	*3.4E-75*	*6.1E-67*	0.021	1	0.0056
cytosolic small ribosomal subunit	0.059	0.0013	0.26	*1.5E-41*	*1.2E-47*	*4.6E-42*	0.073	1	0.03
ribosome	1	0.39	0.3	*2.7E-09*	*6.2E-14*	*3.7E-09*	1	1	0.62

*For each module in each dataset analyzed, an EASE analysis [25] was performed by using Fisher's exact test and the resulting p-values were placed into the table based on the prevalence of the GO (Gene Ontology) term being searched for. The most significant enrichments for a given term are indicated in italics

**Table 2:** Summary of tests for network predictive power

**Module name**	**DNA Dmg**		**Env Stress**		**Cell Cycle**	
	
	**Ess Test**	**Cons Test**	**Ess Test**	**Cons Test**	**Ess Test**	**Cons Test**
Entire Network	0.23 1.0E-39	-0.15 1.3E-19	0.21 1.0E-39	-0.22 1.4E-39	0.2 1.0E-39	-0.18 4.0E-29
rRNA proc	0.2 6.3E-5	-0.1 0.049	0.28 1.0E-39	-0.091 0.0066	0.22 0.00011	-0.23 8.3E-5
Prot Synth	-0.12 0.014	-0.07 0.15	-0.16 0.0035	-0.042 0.46	0.019 0.7	-0.099 0.055
Ubiq	0.41 1.8E-10	-0.28 2.9E-5	0.49 0.0022	-0.16 0.34	-0.044 0.44	-0.032 0.59

*Spearman correlation and associated p-values for each network and module tested
